# Damage of polyesters by the atmospheric free radical oxidant NO_3_^•^: a product study involving model systems

**DOI:** 10.3762/bjoc.9.225

**Published:** 2013-09-20

**Authors:** Catrin Goeschen, Uta Wille

**Affiliations:** 1 ARC Centre of Excellence for Free Radical Chemistry and Biotechnology, School of Chemistry and Bio21 Institute, The University of Melbourne, 30 Flemington Road, Parkville, VIC 3010, Australia

**Keywords:** environmental oxidants, free radicals, nitrate radicals, polyester degradation, product studies, reaction mechanisms

## Abstract

Manufactured polymer materials are used in increasingly demanding applications, but their lifetime is strongly influenced by environmental conditions. In particular, weathering and ageing leads to dramatic changes in the properties of the polymers, which results in decreased service life and limited usage. Despite the heavy reliance of our society on polymers, the mechanism of their degradation upon exposure to environmental oxidants is barely understood. In this work, model systems of important structural motifs in commercial high-performing polyesters were used to study the reaction with the night-time free radical oxidant NO_3_^•^ in the absence and presence of other radical and non-radical oxidants. Identification of the products revealed ‘hot spots’ in polyesters that are particularly vulnerable to attack by NO_3_^•^ and insight into the mechanism of oxidative damage by this environmentally important radical. It is suggested that both intermediates as well as products of these reactions are potentially capable of promoting further degradation processes in polyesters under environmental conditions.

## Introduction

Polymers are without doubt the most important industrial materials, which have benefited our society in numerous ways. Improving the performance of polymers by making them long lasting and durable is therefore highly desirable not only for the consumer but also for the environment, because expensive waste removal strategies can be avoided or at least reduced. The most important way to improve polymer longevity is a detailed knowledge of the mechanism by which they undergo degradation upon exposure to the environment. It is quite surprising that, despite the heavy reliance of our society on polymeric materials, the chemical mechanism of polymer degradation is by far not fully understood.

It has generally been assumed that polymer degradation involves a radical-mediated autoxidation mechanism, which propagates through hydrogen abstraction by an intermediate peroxyl radical ROO^•^. Although this autoxidation mechanism was initially proposed only for a limited number of polymers that contain activated allylic hydrogen atoms (for example rubber materials) [1–5], it has been universally adapted as general mechanism for polymer degradation. However, recent comprehensive high-level theoretical studies by Coote et al. clearly revealed that polymers possessing only saturated alkyl chains, for example polyesters, will not propagate autoxidation, particularly because the ROO–H bond-dissociation energy (BDE) is usually less than the BDE for unactivated R–H bonds [6].

**Scheme 1:**
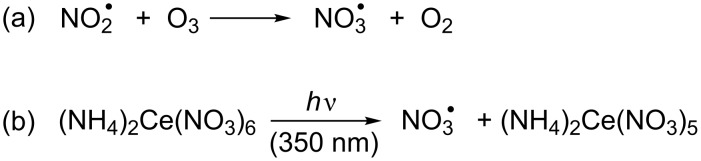
Generation of NO_3_^•^ (a) in the atmosphere, (b) under experimental conditions.

Polymer surface coatings, which are widely used in the building, automotive and aircraft industries to protect the underlying material from degradation, are commonly high-performing polyesters, which are exposed to significant environmental stress, in particular high temperatures, humidity and UV irradiation. These materials are in direct contact with the troposphere, which is the lowest part of the atmosphere and a highly oxidizing environment. While the oxidation power during daytime can be assigned to the presence of hydroxyl radicals, HO^•^, the highly electrophilic nitrate radical, NO_3_^•^, is responsible for the tropospheric transformation processes at night. NO_3_^•^, which is formed through reaction of the atmospheric pollutants nitrogen dioxide, NO_2_^•^, with ozone, O_3_ (Scheme 1a) [7–8], reacts with organic compounds through various pathways, such as hydrogen abstraction (HAT) and addition to π systems. Most importantly, NO_3_^•^ is one of the strongest free-radical oxidants known [*E*(NO_3_^•^/NO_3_^−^) = 2.3–2.5 V vs NHE] [9], and recent product studies by us revealed that NO_3_^•^ readily damages aromatic amino acids and pyrimidine nucleosides through an oxidative pathway [10–13]. Thus, the ease by which model compounds of biologically important macromolecules are attacked by NO_3_^•^ leads inevitably to the question, how resistant synthetic polymers are towards oxidative damage by this environmental free-radical species, in particular in conjunction with other atmospheric radical and non-radical oxidants, which are in direct contact with these materials. Is it possible that such reactions could lead to structural modifications in the polymer that may render the material more susceptible to further damage, for example through photodegradation and/or autoxidation? To our knowledge, the role of environmental free-radical oxidants as mediator of polymer degradation has barely been assessed so far.

In light of this, we have now performed the first product study of the reaction of NO_3_^•^ with model substrates relevant to the polymeric structures in high-performing polyesters in the presence and absence of other oxidants, in particular NO_2_^•^, O_2_ and O_3_. This work not only reveals new insight into the degradation mechanism in polyesters upon exposure to important environmental oxidants, but it also enables identification of vulnerable sites (‘hot spots’) in the polymer, which could open up new pathways to polyester degradation under environmental conditions that have not been considered before. This study might therefore be regarded as a first step on a long journey towards a revised mechanistic scheme for polymer degradation, which is crucial for the development of improved materials.

## Results and Discussion

### Experimental conditions

**Figure 1:**
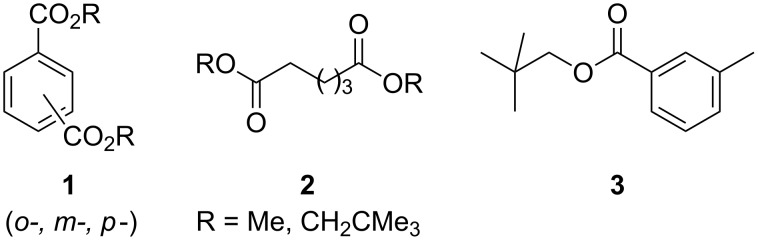
Polyester-model systems studied in this work.

The compounds that served in this work as models for substructures typically found in surface-coating polyesters are shown in Figure 1. These comprise aromatic moieties, such as phthalic and benzoic esters **1** and **3**, respectively, as well as aliphatic diesters of type **2**. The esters were used as methylates or neopentylates, where the latter provided a simplified model for diesters of neopentyl glycol, which is the commonly used diol component in such polyesters.

All experiments were performed in solution, using two different methods to produce NO_3_**^•^** in situ in the presence of the respective substrate **1**–**3**. In experiments where NO_3_**^•^** was used in the absence of other radical and non-radical oxidants, NO_3_**^•^** was generated at room temperature from cerium(IV) ammonium nitrate (CAN) through photo-induced electron transfer at an irradiation wavelength of λ = 350 nm (Scheme 1b) [11–13].

In a typical experiment, the polyester-model substrate and four equivalents of CAN were dissolved in acetonitrile and the solutions degassed by sonicating under a continuous argon stream, followed by irradiation and aqueous work-up. Control experiments performed under exclusion of light showed no reaction, which ensured that the observed products indeed resulted from the reaction involving NO_3_**^•^** and not from CAN, which is also an oxidizing agent [*E*^0^(Ce^4+^/Ce^3+^) = 1.61 V vs. NHE] [14].

In another set of experiments, NO_3_**^•^** was obtained through the reaction shown in Scheme 1a, where to a solution of the respective polyester-model substrate in anhydrous dichloromethane at 10 °C an excess of liquid NO_2_**^•^** was added and ozonized O_2_ was bubbled through the mixture at a low flow rate, followed by aqueous work-up. Through control experiments it was revealed that none of the various polyester-model compounds reacted to a noticeable extent with NO_2_**^•^** or ozonized O_2_ in isolation. Reactions with this NO_3_**^•^** source in acetonitrile gave identical products to those in dichloromethane. However, additional products were also obtained in small amounts, which could not be identified. It is possible that these resulted from hydrolysis of dinitrogen pentoxide, N_2_O_5_, which is formed through reversible recombination of NO_2_**^•^** with NO_3_**^•^**, by trace amounts of water present in acetonitrile, but this was not further explored. By performing the radical reactions in dichloromethane, potentially interfering reactions involving the solvent, which could complicate the reaction outcome and mechanic considerations, were avoided. It should also be noted that NO_2_**^•^** is in equilibrium with its dimer dinitrogen tetroxide, N_2_O_4_. In solution the NO_2_**^•^**/N_2_O_4_ equilibrium constant favours the dimer [15], and N_2_O_4_ can be oxidized with O_3_ to give N_2_O_5_. Gas-phase kinetic studies revealed that N_2_O_5_ reacts with unsaturated compounds several orders of magnitude slower than NO_3_**^•^** [16] and does not readily nitrate deactivated aromatic compounds in solution [17].

In all experiments we have used NO_3_**^•^** in excess in order to obtain sufficient amounts of material to enable product separation by preparative HPLC using UV detection at wavelengths of λ = 214 and 230 nm and identification by spectroscopic characterization. Details are given in the Experimental section. HPLC chromatograms of the relevant raw reaction mixtures are shown in Supporting Information File 1. Although under natural conditions NO_3_**^•^** will be present in much lower concentrations compared to the polyester, our experimental procedure ensured that vulnerable sites in the polyester-model systems could be located with certainty. Due to the repeated purification by HPLC, yields could not be obtained for any of these reactions. However, since this study is aimed at obtaining insight into the nature of the products in order to qualitatively assess how such chemical modifications might affect polymer stability under environmental conditions, exact yields are not required. It is reasonable to assume that only very few damaged sites are initially required in the polyester to promote further degradation on a large scale through chain and other processes.

### Reaction of polyester-model compounds **1**–**3** with NO_3_^•^ from CAN photolysis

**Scheme 2:**
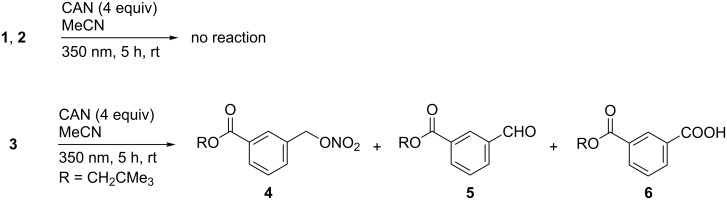
Products of the reaction of polyester model compounds **1**–**3** with NO_3_^•^ in the absence of other radical and non-radical oxidants.

Study of the products formed in the reaction of NO_3_**^•^** obtained from CAN photolysis provides the opportunity to gain insight into the mechanism of oxidative damage in the absence of other radical and non-radical oxidants. In Scheme 2 the products of the reaction of the polyester-model compounds **1**–**3** with NO_3_**^•^** are shown.

It was interesting to note that no reaction occurred with the isomeric phthalates **1** and the adipic acid derivatives **2**. In the case of the former this could be explained by the fact that the aromatic ring is very deactivated due to the two electron-withdrawing ester substituents, so that oxidative electron transfer (ET) by NO_3_**^•^** is not possible. Also, NO_3_**^•^** induced HAT from the ester, particularly the neopentyl moiety, which is a potential pathway that should most likely occur at the methylene groups α to the ester oxygen atom [18–20], is apparently not a feasible pathway. This finding is of potential relevance for the autoxidation mechanism, which proposes hydrogen abstraction by ROO^•^ as propagating step. Thus, although NO_3_**^•^** is not only much more reactive than ROO**^•^** [7–8], and the BDE for the O_2_NO–H bond is with 427 kJ mol^−1^ also considerably higher than that of the ROO–H bond (which is about 360 ± 20 kJ mol^−1^) [21], the fact that no hydrogen abstraction from the ester units was observed in the reactions with NO_3_**^•^** demonstrates that saturated alkyl groups are quite inert to radical attack.

On the other hand, in the case of neopentyl ester of *m*-toluic acid (**3**), which differs from the phthalates by replacement of one ester group by a σ-donating methyl group, reaction with NO_3_**^•^** leads to selective oxidative modification of the methyl side chain, while a reaction at the ester moiety was, again, not observed. Analytical HPLC of the raw reaction mixture recorded at λ = 230 nm revealed besides unreacted starting material **3** (which was identified by comparison with an authentic sample but not isolated), nitrate **4**, aldehyde **5** and carboxylic acid **6** as most important products (see Supporting Information File 1). Other products were formed in too minor amounts to enable isolation. Further, HPLC analysis revealed that shorter reaction times or a smaller excess of CAN shifted the product ratio towards the nitrate **4** at expense of the higher oxidized products **5** and **6** (data not shown).

**Scheme 3:**
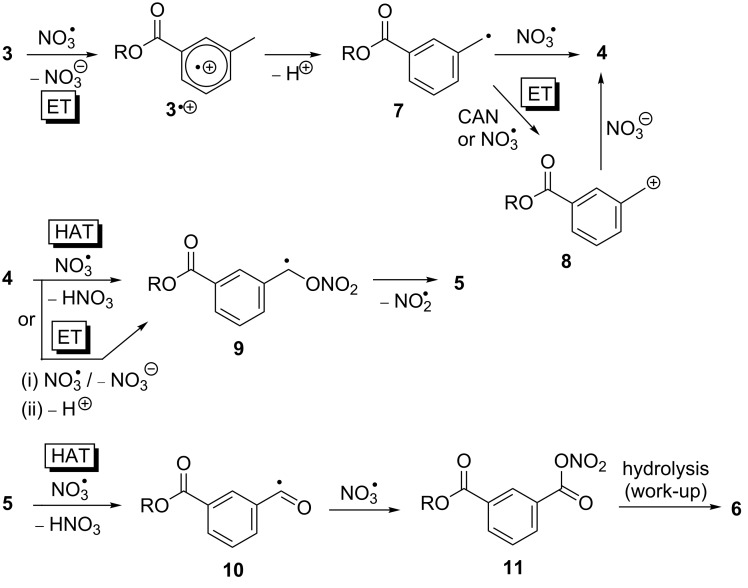
Proposed mechanism for the reaction of *m*-toluic acid neopentyl ester (**3**) with NO_3_^•^ in the absence of radical and non-radical oxidants.

The observed side-chain oxidation in **3** by NO_3_**^•^** is similar to the outcome of the reaction of thymidine nucleosides with NO_3_**^•^**, where oxidative transformation of the methyl substituent in the heterocyclic base occurs exclusively [13]. Concentration–time profiles revealed for the latter reactions that formation of a nitrate occurs first, which is converted to an aldehyde and subsequently into a carboxylic acid [13]. It is not unreasonable to assume that such a step-wise oxidation also occurs in the reaction involving **3**, which could be rationalized by the mechanism shown in Scheme 3.

Because of the high oxidation power of NO_3_^•^, it is proposed that the reaction is initiated by ET at the aromatic ring through an addition–elimination pathway, as has been suggested from time-resolved transient spectroscopic studies for the reaction of NO_3_^•^ with alkylaromatic compounds [22–23]. In the absence of any reactants the resulting radical cation **3**^•+^ undergoes deprotonation to give benzyl radical **7**, in analogy to the mechanism of the NO_3_^•^-induced oxidation of aromatic amino acids and nucleosides [10–13]. This mechanism is supported by findings by Steenken et al., who showed that in the reaction of alkylaromatic compounds with NO_3_^•^ ET and deprotonation can occur practically in a concerted fashion in the case of highly electron-rich arenes, while in the case of less activated alkylaromatic compounds the intermediate radical cation has a lifetime on the nanosecond time scale [23]. It was further demonstrated that deprotonation of arylradical cations is accelerated by nitrate (NO_3_**^−^**) that is present in the reaction system as ‘byproduct’ of the oxidation process and as ligand in CAN, and which acts as a Brønsted base [23]. It is important to note that the formation of radical intermediate **7** could principally also occur in one step through NO_3_^•^-induced benzylic HAT in **3** (not shown). However, it appears from the outcome of the reactions with the neopentyl derivatives of **1** and **2** that HAT by NO_3_^•^ is not competitive with NO_3_^•^-induced ET in these systems [7–8,24–25]. An initial ET step and formation of an intermediate radical cation **3**^•+^ is further supported by the outcomes of the reaction of **3** with NO_3_^•^ in the presence of NO_2_^•^, which will be outlined below.

Formation of nitrate **4** could principally occur via two different pathways, e.g. through direct trapping of **7** by NO_3_^•^ or in a two-step process by first NO_3_^•^ or CAN-induced ET, followed by quenching of the resulting benzyl cation **8** through ligand exchange from CAN. Although the nature of the intermediate was not further explored in this work, our previous experiments involving thymidines provided strong indications that the reaction likely involves a cationic intermediate [13].

Conversion of the nitrate ester **4** into the aldehyde **5** could proceed through either an intermediate benzyl radical **9**, which could be formed through a direct HAT by NO_3_^•^ [26], or through a sequential ET–deprotonation pathway in analogy to the initial reaction step. The labile O–NO_2_ bond in **9** is expected to undergo rapid β-scission to give aldehyde **5** with release of NO_2_^•^ [27–28]. The latter is too unreactive to initiate a radical process in this system, which has been confirmed through independent control experiments.

Oxidation of aldehyde **5** to the carboxylic acid **6** under the experimental conditions could by initiated through abstraction of the aldehyde hydrogen atom by NO_3_^•^ [29], followed by trapping of the resulting acyl radical **10** by NO_3_^•^ to give the mixed anhydride **11**, which could be hydrolysed to the acid **6** during aqueous work-up and/or purification by HPLC.

The mechanism in Scheme 3 shows that more than one equivalent of NO_3_^•^ is required to produce the observed products **4**–**6**. Such multiple attacks seem unlikely under environmental conditions, where [NO_3_^•^] is low [7–8]. However, from the previous work on NO_3_^•^-induced oxidative damage of biological molecules, it appears that an already damaged compound is more prone to attack by another NO_3_^•^ than an undamaged substrate [11–13].

### Reaction of polyester-model compounds **1**–**3** with NO_3_^•^ from NO_2_^•^/O_3_

Under environmental conditions, however, NO_3_**^•^** is not an isolated reactant, but is always accompanied by other radicals and non-radical oxidants, such as NO_2_**^•^**, O_3_, and O_2_, respectively, which principally could become involved in these reactions through trapping of reactive intermediates. Thus, in order to explore the role of such additional reactants on the outcome of NO_3_**^•^**-induced oxidative damage of polyester-model compounds, we have used the reaction in Scheme 1a to produce NO_3_**^•^**.

Similar to the reaction with NO_3_**^•^** in isolation, no reaction of NO_3_**^•^** with phthalates **1** and adipic esters **2** was observed in the presence of NO_2_**^•^**, O_3_, and O_2_, which is a further confirmation for the low reactivity of saturated alkyl chains. On the other hand, the reaction with the *m*-toluic acid ester **3** was very fast. According to the HPLC spectrum of the raw reaction mixture (see Supporting Information File 1), the starting material was completely consumed and considerably more products were obtained in the presence of NO_2_**^•^**, O_3_, and O_2_ compared to the reaction of NO_3_**^•^** in isolation.

**Scheme 4:**
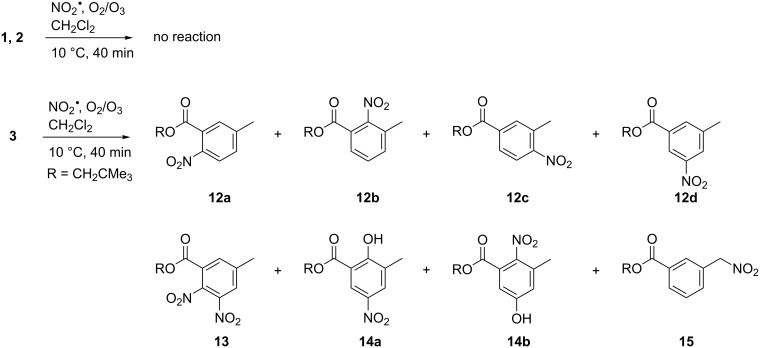
Products of the reaction of polyester-model compounds **1**–**3** with NO_3_^•^ in presence of NO_2_^•^, O_3_, and O_2_.

**Scheme 5:**
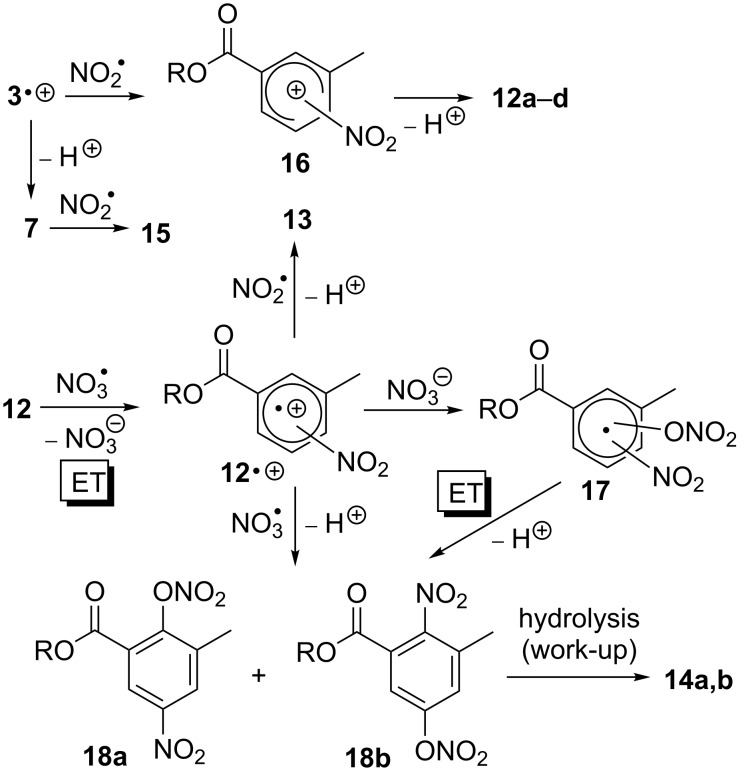
Proposed mechanism for the reaction of *m*-toluic acid neopentyl ester (**3**) with NO_3_^•^ in presence of NO_2_^•^, O_3_ and O_2_.

The main reaction pathways lead to products possessing a nitroaromatic ring, such as the isomeric mono-nitroaromatic compounds **12a**–**d**, the dinitrated product **13** and two isomeric species **14a**,**b**, which carry both a nitro and a hydroxy substituent (Scheme 4). The nitro compound **15** appears to be the only product that results from oxidative modification of the methyl substituent at the aromatic ring. HPLC analysis indicated that additional products were formed in this reaction (see Supporting Information File 1), but their amounts were too small to enable isolation and identification. The proposed mechanism leading to the various products **12**–**15** is outlined in Scheme 5.

Similar to the mechanism shown in Scheme 3, initial ET should lead to the radical cation **3^•+^**. However, in contrast to the reaction with NO_3_**^•^** in isolation, where benzylic deprotonation occurred exclusively, in the presence of excess NO_2_**^•^** the radical cation **3^•+^** is trapped prior to deprotonation to form the isomeric σ-complexes **16** [30]. The aromatic ring is restored through loss of a proton, which leads to the nitroaromatic products **12a**–**d**. The proposed competition in radical cation **3^•+^** between trapping by NO_2_**^•^** and benzylic deprotonation is supported by the fact that the nitromethylene compound **15** is obtained as byproduct in this reaction. The latter likely results from recombination of NO_2_**^•^** with benzyl radical **7**, which is obviously formed in small amounts. Thus, in contrast to the reaction of **3** with NO_3_**^•^** in isolation, formation of stable reaction products in the presence of NO_2_**^•^**, O_3_ and O_2_, such as the nitroaromatic compounds **12**, requires only one equivalent of NO_3_**^•^** and should readily occur even at low atmospheric [NO_3_**^•^**].

Formation of the tetrasubstituted products **13** and **14** proceeds likely through a second NO_3_**^•^**-induced ET in the mono-nitrated compounds **12a**,**b**, where the intermediately formed radical cation **12^•+^** can be trapped by NO_2_**^•^** to give the dinitro compound **13** after deprotonation. This mechanism is supported by previous findings in the reaction of aromatic amino acids with NO_3_**^•^** in the presence of NO_2_**^•^**, O_3_, and O_2_, where it was shown that dinitrated products result from a step-wise nitration of the aromatic ring [10]. On the other hand, to our knowledge, formation of hydroxylated products of type **14** in the reaction of NO_3_**^•^** with aromatic compounds is unprecedented. We propose that these compounds result from hydrolysis of the corresponding nitrates **18** during work-up and/or HPLC purification. Potential pathways to the latter could involve either trapping of the radical cation **12^•+^** by NO_3_**^•^**, followed by deprotonation, or recombination of **12^•+^** with NO_3_^−^ (the byproduct of the NO_3_**^•^**-induced ET). The resulting radical adduct **17** could be oxidized in a subsequent step by either NO_3_**^•^** or NO_2_**^•^** [*E*(NO_2_**^•^**/NO_2_^−^) = 1.04 V vs NHE] [31], which is followed by deprotonation to restore the aromatic system.

## Conclusion

We have shown for the first time that certain aromatic moieties in commercial polyesters (e.g. alkylated benzoic acid derivatives of type **3**) are vulnerable to damage by the environmental free-radical oxidant NO_3_^•^. The reaction is most likely initiated by ET to give a highly reactive aryl radical cation intermediate **3**^•^**^+^**, whose fate depends strongly on the reaction conditions. In the absence of radical-trapping agents, in particular NO_2_^•^, benzylic deprotonation is the exclusive pathway that ultimately leads to oxidative functionalization of the alkyl side chain through formation of nitrates **4**, aldehydes **5** and carboxylic acids **6**. In this work we have not specifically explored the role of O_2_ on the reaction outcome, but our recent studies on the NO_3_^•^-induced oxidative damage in thymidines showed that any residual O_2_ present in the system solely accelerates production of the higher oxidized compounds **5** and **6**, while no different products are formed [13]. It is reasonable to expect a similar outcome for the reaction of NO_3_^•^ with **3** in the presence of O_2_. On the other hand, when the reaction of NO_3_^•^ with **3** is performed in the presence of NO_2_^•^, benzylic deprotonation in radical cation **3**^•^**^+^** can hardly compete with trapping of the latter by NO_2_^•^, which leads to formation of the isomeric nitroaromatic compounds **12a**–**d** as well as the dinitro and hydroxylated products **13** and **14**, respectively, that result from further NO_3_^•^-induced oxidation of **12**. An additional, however only minor pathway yields the nitromethylene compound **15**, which is formed via benzyl radical **7**. Although we have not studied the nature of the reactive intermediates formed in these reactions, it is difficult to rationalize formation of the ring-substituted products **12**–**14** through a mechanism that involves benzylic HAT by NO_3_^•^. The reaction must therefore be initiated by oxidation of the aromatic ring, which is in accordance with literature findings [22–23].

The different outcome of the reaction of NO_3_^•^ with **3** under the various conditions could be explained by the different concentration of NO_2_^•^ and NO_3_^−^ in these systems. Thus, CAN photolysis generates NO_3_^•^ in the presence of excess NO_3_^−^, which acts as Brønsted base and mediates deprotonation of the initial radical cation **3**^•^**^+^** to give benzyl radical **7** [23], followed by transformation to the products **4**–**6**. In the NO_2_^•^/O_3_ system, on the other hand, [NO_3_^−^] = [**3**^•^**^+^**] and deprotonation in **3**^•^**^+^** cannot complete with its trapping by excess NO_2_^•^, which leads to the nitroaromatic species **12**–**14**.

In contrast to the high reactivity of the aromatic ring in **3**, phthalate-building blocks as well as ester moieties possessing only saturated alkyl chains appear to be inert to attack by NO_3_^•^ through either ET or HAT, respectively, under the various conditions explored. Our observation that NO_3_^•^-induced HAT in the ester moieties does not occur, although NO_3_^•^ is much more reactive than ROO^•^ and the O_2_NO–H bond is considerably stronger than the ROO–H bond, could be taken as indication that an autoxidation mechanism involving ROO^•^ as chain carrier cannot operate in intact polyesters with saturated alkyl chains, which is in support of the theoretical findings by Coote et al. [6].

None of the various polyester-model compounds explored in this work reacted with NO_2_^•^ and O_3_ in isolation. However, this outcome is not unexpected, since the reactivity of NO_2_^•^ is much lower than that of NO_3_^•^. In particular, the oxidation power of NO_2_^•^ is not sufficient to induce ET in deactivated aromatic compounds [31]. Likewise, although O_3_ is a strong oxidant, it does not react via ET transfer. Rapid reactions are only expected for π systems, such as alkenes, which are not present in intact polyester materials (however, it should be noted that these structural motifs may be formed in the polymer through degradation processes).

What are the potential implications of NO_3_^•^-induced oxidative damage in aromatic building blocks for polyester stability? Although there are no experimental data available yet, it is possible to make some predictions from the outcome of this work, which can be used to guide future studies on polyester stability upon exposure to the environment. It is important to realize that under environmental conditions only few sites of initial damage are required to trigger degradation of the polymer material on a large scale. Identification of the reaction products using simplified model systems enables to obtain some general insight into the mechanism of radical-induced oxidative damage in these materials. Thus, in the reaction of the aromatic ester **3** with NO_3_^•^ it could be speculated that both intermediates as well as products could principally promote further damage in the polymer. For example, the radical cation **3**^•^**^+^** is itself a highly oxidizing intermediate, which could, when embedded in the polyester matrix, induce an ET cascade across the polymer involving aromatic moieties, where oxidative damage may end up at positions remote from the initial site of attack. The benzyl radical **7** resulting from deprotonation in **3**^•^**^+^** on the other hand, could be trapped by O_2_ and be involved as chain carrier in subsequent transformations that lead to degradation.

Of the various products formed in the reaction of NO_3_^•^ with **3** under the different reaction conditions, in particular the aldehyde **5** and the nitroaromatic species **12**–**14** are expected to be photochemically active compounds. Exposure of the carbonyl or nitro moieties to UV light leads to photoexcited intermediates, which are strong hydrogen-atom abstractors in Norrish-type II photoreactions [32–33]. In the polymer matrix, where the various polyester chains are tightly packed, both intra- and interstrand reactions are likely to occur, such as photo-induced hydrogen abstractions, which could provide pathways to C-radicals in unactivated alkyl chains that would usually be inert to attack by peroxyl radicals.

To conclude, this work provides strong indications for a number of so far unexplored pathways that could promote degradation of high-performing polyesters under environmental conditions. It is obvious that detailed kinetic data and product analyses from exposure studies involving both simple as well as more complex model systems, including melamine cross-linker moieties, are required (for example from smog chamber experiments), to obtain further insight into the role of environmental free-radical oxidants, such as NO_3_^•^ and HO^•^, in promoting polyester degradation.

## Experimental

### General procedures

The irradiations were performed under a continuous gas flow (argon) in a Rayonet photochemical reactor (λ = 350 nm). Before the irradiations, residual oxygen was removed from the reaction mixture by bubbling argon through the solution while sonicating. ^1^H and ^13^C NMR spectra were taken on a Varian Unity Inova 500 spectrometer [500 MHz (^1^H), 125 MHz (^13^C)] or on an Agilent MR 400 spectrometer [400 MHz (^1^H), 100 MHz (^13^C)] in deuterated DMSO. If necessary the assignment of the chemical shifts was confirmed by utilising 2D NMR techniques. GC–MS (EI, 70 eV) analysis was run on an Agilent 7890A GC/5975C MSD, column from SUPELCO 30 m, 0.32 mm ID, 0.25 μm film thickness fused silica capillary column, using the temperature program 70_5_ → 250_17_ heating rate 5 ºC min**^−^**^1^ (40 min in total). HRMS was conducted by ionising the samples via ESI into a Thermo-Finnigan LTQ FT–ICR hybrid mass spectrometer or an Agilent 6520 LC/Q-TOF mass spectrometer with an electrospray ionizing source coupled to an Agilent 1100 LC system equipped with a variable wavelength detector. The crude products were purified by reversed-phase HPLC (Phenomenex C18, 150 × 21.2 mm, 5 micron, preparative column, 8 mL min**^−^**^1^) using an Agilent 1100 LC system equipped with a variable wavelength detector by running a gradient from 50% water in acetonitrile to 100% acetonitrile within 2–3 hours. UV detection was performed at λ = 214 and 230 nm. Purity was assessed by analytical RP HPLC on an SGE Protecol C18 5 μM 250 × 4.6 mm column.

### Reactions with NO_3_^•^ from CAN photolysis

In a typical experiment 1.0 mmol of the model substrate and 4.0 mmol of CAN were dissolved in 5 and 95 mL of absolute acetonitrile, respectively, and the individual solutions degassed by sonicating under a continuous argon stream. The solutions were combined and irradiated (λ = 350 nm) for a period of 5 h at room temperature. The reaction was quenched by addition of brine (50 mL) and water (50 mL) and extracted with ethyl acetate. The combined organic fractions were dried (MgSO_4_) and the solvent removed in vacuum. The crude product mixture was separated by reversed-phase HPLC.

Neopentyl 3-(nitratomethyl)benzoate (**4**): ^1^H NMR (DMSO-*d*_6_, 500 MHz) δ 8.08 (td, = 1.8, 0.6 Hz, 1H), 8.01 (ddd, *J =* 7.8, 1.8, 1.2 Hz, 1H), 8.75 (dtd, *J =* 7.6, 1.2, 0.6 Hz, 1H), 7.60 (td, *J =* 7.7, 0.5 Hz, 1H), 5.66, (s, 2H), 3.99 (s, 2H), 0.99 (s, 9H); ^13^C NMR (DMSO-*d*_6_, 125 MHz) δ 165.7, 134.5, 133.7, 130.7, 130.3, 130.2, 129.9, 74.9, 74.2, 31.8, 26.7; HRMS (*m*/*z*): calcd for C_13_H_17_NO_5_ + H, 268.1185; found, 268.1181; HRMS (*m*/*z*): calcd for C_12_^13^CH_17_NO_5_ + H, 269.1219; found, 269.1212.

Neopentyl 3-formylbenzoate (**5**): ^1^H NMR (DMSO-*d*_6_, 500 MHz) δ 10.10 (s, 1H), 8.45 (td, *J* = 1.7, 0.5 Hz, 1H), 8.27 (ddd, *J* = 7.7, 1.8, 1.2 Hz, 1H), 8.17 (td, *J* = 7.7, 1.5 Hz, 1H), 7.77 (td, *J* = 7.7, 0.5 Hz, 1H), 4.02 (s, 2H), 1.00 (s, 9H); ^13^C NMR (DMSO-*d*_6_, 125 MHz) δ 192.8, 164.9, 136.5, 134.5, 133.6, 130.7, 130.0, 129.9, 73.9, 31.4, 26.2; HRMS (*m*/*z*): calcd for C_13_H_16_O_3_ + H: 221.1178. Found: 221.1170; HRMS (*m*/*z*): calcd for C_12_^13^CH_16_O_3_ + H, 222.1211; found, 222.1204.

Neopentyl 3-carboxylbenzoate (**6**): ^1^H NMR (DMSO-*d*_6_, 500 MHz) δ 13.28 (s(br), 1H), 8.49 (td, *J* = 1.8, 0.5 Hz, 1H), 8.19 (d, *J* = 1.7 Hz, 1H), 8.18 (dd, *J* = 1.8, 0.5 Hz, 1H), 7.66 (td, *J* = 7.8, 0.6 Hz, 1H), 4.00 (s, 2H), 0.98 (s, 9H); ^13^C NMR (DMSO-*d*_6_, 125 MHz) δ 166.9, 165.4, 134.2, 133.6, 131.9, 130.7, 130.1, 129.9, 74.2, 31.8, 26.7; HRMS (*m*/*z*): calcd for C_13_H_16_O_4_ + Na, 259.0946; found, 259.0940; HRMS (*m*/*z*): calcd for C_12_^13^CH_16_O_4_ + Na, 260.0980; found, 260.0974.

### Reactions with NO_3_^•^ generated from NO_2_^•^/O_3_

In a typical experiment 0.5 mL liquid NO_2_^•^ (15 mmol) was added to 1.00 mmol of the model substrate in anhydrous dichloromethane (15 mL) at 10 °C, and ozonised O_2_ was bubbled through the mixture at a low flow rate. After 40 min the reaction was quenched by addition of 10 mL aq NaHCO_3_, the phases were separated and the aqueous phase extracted with dichloromethane. The combined organic fractions were dried over MgSO_4_, concentrated and the reaction products isolated and purified by repeated preparative HPLC. It was not possible to state the exact [NO_2_^•^] in these experiments, since an indeterminable amount evaporated prior to its reaction with O_3_.

Neopentyl 5-methyl-2-nitrobenzoate (**12a**): ^1^H NMR (DMSO-*d*_6_, 500 MHz) δ 7.95 (d, *J* = 8.3 Hz, 1H), 7.65 (dd, *J* = 1.8, 0.9 Hz, 1H), 7.60 (ddd, *J* = 8.3, 1.9, 0.9 Hz, 1H), 3.94 (s, 2H), 2.45 (s, 3H), 0.92 (s, 9H); ^13^C NMR (DMSO-*d*_6_, 100 MHz) δ 165.4, 145.2, 133.3, 130.5, 127.1, 124.6, 75.5, 31.6, 26.5, 21.2. The signal of the carbon atom carrying the nitro substituent (C-2) could not be observed; MS (EI, 70 eV) *m*/*z* (%): 251.1 (1) [M^+^], 164.1 (100) [M^+^ − OCH_2_C(CH_3_)_3_], 57.2 (31) [C(CH_3_)_3_^+^]; IR (cm^−1^) ν: 2960, 1730, 1527, 1367, 1347, 1257,1200, 1072, 833.

Neopentyl 3-methyl-2-nitrobenzoate (**12b**): ^1^H NMR (DMSO-*d*_6_, 500 MHz) δ 7.89 (d, *J* = 7.7 Hz, 1H), 7.76 (d, *J* = 7.6 Hz, 1H), 7.67 (d, *J* = 7.7 Hz, 1H), 3.95 (s, 2H), 2.30 (s, 3H), 0.94 (s, 9H); ^13^C NMR (DMSO-*d*_6_, 100 MHz) δ 163.6, 136.7, 131.4, 130.7, 129.2, 123.1, 75.5, 31.6, 26.5, 16.8. The signal of the carbon atom carrying the nitro substituent (C-2) could not be observed; MS (EI, 70 eV) *m*/*z* (%): 251.1 (1) [M^+^], 164.1 (100) [M^+^ − OCH_2_C(CH_3_)_3_], 57.1 (40) [C(CH_3_)_3_^+^]; IR (cm^−1^) ν: 2958, 1727, 1533, 1369, 1287.

Neopentyl 3-methyl-4-nitrobenzoate (**12c**): ^1^H NMR (DMSO-*d*_6_, 500 MHz) δ 8.08 (d, *J* = 8.5 Hz, 1H), 8.06 (d, *J* = 1.5 Hz, 1H), 8.00 (dd, *J* = 8.5, 1.9 Hz, 1H), 4.03 (s, 2H), 2.56 (s, 3H), 1.01 (s, 9H); ^13^C NMR (DMSO-*d*_6_, 100 MHz) δ 164.7, 152.2, 138.8, 133.7, 133.5, 128.4, 125.3, 74.6, 31.8, 26.7, 19.5; MS (EI, 70 eV) *m*/*z* (%): 251.1 (4) [M^+^], 164.1 (100) [M^+^ − OCH_2_C(CH_3_)_3_], 57.1 (90) [C(CH_3_)_3_^+^]; IR (cm^−1^) ν: 2960, 1723, 1526, 1367, 1259,1192, 118, 1026, 839, 735.

Neopentyl 5-methyl-3-nitrobenzoate (**12d**): ^1^H NMR (DMSO-*d*_6_, 500 MHz) δ 8.45 (m, 1H), 8.35 (ddd, *J* = 2.5, 1.5, 0.8 Hz, 1H), 8.20 (dt, *J* = 1.8, 1.1 Hz, 1H), 4.05 (s, 2H), 2.53 (s, 3H), 1.01 (s, 9H); ^13^C NMR (DMSO-*d*_6_, 100 MHz) δ 164.5, 148.4, 141.7, 136.0, 131.6, 128.4, 121.3, 74.7, 31.8, 26.7, 21.0; MS (EI, 70 eV) *m*/*z* (%): 251.1 (1) [M^+^], 164.1 (91) [M^+^ − OCH_2_C(CH_3_)_3_], 57.1 (100) [C(CH_3_)_3_^+^]; IR (cm^−1^) ν: 2960, 1656, 1541, 1289.

Neopentyl 5-methyl-2,3-dinitrobenzoate (**13**): ^1^H NMR (DMSO-*d*_6_, 500 MHz) δ 8.14 (t, *J* = 1.7 Hz, 1H), 8.05 (dt, *J* = 7.8, 1.4 Hz, 1H), 7.79 (ddd, *J* = 7.7, 1.8, 1.3 Hz, 1H), 5.88 (s, 2H), 4.01 (s, 2H), 1.01 (s, 9H); ^13^C NMR (DMSO-*d*_6_, 125 MHz) δ 163.3, 145.8, 140.7, 138.6, 133.9, 132.6, 124.7, 75.3, 31.9, 26.6, 17.0; MS (EI, 70 eV) *m/z* (%): 296.1 (1) [M^+^], 209.0 (34) [M^+^ − OCH_2_C(CH_3_)_3_], 57.1 (100) [C(CH_3_)_3_^+^]; IR (cm^−1^) ν: 2958, 1724, 1548, 1347, 756.

Neopentyl 2-hydroxy-3-methyl-5-nitrobenzoate (**14a**): ^1^H NMR (DMSO-*d*_6_, 500 MHz) δ 11.57 (s(br), 1H), 8.48 (d, *J* = 2.9 Hz, 1H), 8.35 (dd, *J* = 2.7, 1.2 Hz, 1H), 4.11 (s, 2H), 2.30 (s, 3H), 1.02 (s, 9H); ^13^C NMR (DMSO-*d*_6_, 100 MHz) δ 168.5, 164.2, 130.8, 129.0, 123.7, 112.4, 75.1, 31.8, 26.6, 15.9. The signal of the carbon atom carrying the nitro substituent (C-5) could not be observed. MS (EI, 70 eV) *m*/*z* (%): 267.1 (24) [M^+^], 180.0 (100) [M^+^ − OCH_2_C(CH_3_)_3_], 57.1 (25) [C(CH_3_)_3_^+^]; IR (cm^−1^) ν: 2966, 1676, 1335, 1173.

Neopentyl 5-hydroxy-3-methyl-2-nitrobenzoate (**14b**): ^1^H NMR (DMSO-*d*_6_, 500 MHz) δ 10.74 (s(br), 1H), 7.12 (dd, *J* = 2.7, 0.6 Hz, 1H), 6.99 (dd, *J* = 2.6, 0.8 Hz, 1H), 3.91 (s, 2H), 2.25 (s, 3H), 0.93 (s, 9H); ^13^C NMR (DMSO-*d*_6_, 100 MHz) δ 164.2, 159.3, 142.4, 133.6, 126.7, 121.5, 114.9, 75.4, 31.6, 26.5, 17.7; MS (EI, 70 eV) *m*/*z* (%): 267.1 (21) [M^+^], 180.0 (100) [M^+^ − OCH_2_C(CH_3_)_3_], 57.1 (69) [C(CH_3_)_3_^+^]; IR (cm^−1^) ν: 2959, 1724, 1532, 1370, 1347, 1333, 1238, 1097.

Neopentyl 3-(nitromethylene)benzoate (**15**): ^1^H NMR (DMSO-*d*_6_, 500 MHz) δ 8.53 (d, *J* = 1.7 Hz, 1H), 8.48 (dd, *J* = 2.1 Hz, 1H), 4.08 (s, 2H), 2.47 (s, 3H), 1.02 (s, 9H); ^13^C NMR (DMSO-*d*_6_, 125 MHz) δ 165.7, 135.9, 131.8, 131.7, 130.7, 130.5, 129.8, 78.7, 74.2, 31.8, 26.7; MS (EI, 70 eV) *m/z* (%): 205.1 (100) [M^+^ − NO_2_], 164.1 (34) [M^+^ − OCH_2_C(CH_3_)_3_], 57.1 (31) [C(CH_3_)_3_^+^]; IR (cm^−1^) ν: 2963, 1720, 1557, 1370, 1282, 1198.

## Supporting Information

File 1 HPLC chromatograms of raw reaction mixtures.
