# Supplementary material for: Damage of polyesters by the atmospheric free radical oxidant NO3•: a product study involving model systems
Source: Beilstein J Org Chem. 2013 Sep 20;9:1907–16. doi: 10.3762/bjoc.9.225 (PMC3817593; doi:10.3762/bjoc.9.225)

Supporting Information

for

Damage of polyesters by the atmospheric free radical oxidant $NO_3^{\bullet}$: a product study involving model systems

Catrin Goeschen and Uta Wille*

Address: ARC Centre of Excellence for Free Radical Chemistry and Biotechnology, School of Chemistry and Bio21 Institute, The University of Melbourne, 30 Flemington Road, Parkville, VIC 3010, Australia

Email: Uta Wille - uwille@unimelb.edu.au

* Corresponding author

HPLC chromatograms of raw reaction mixtures

Reaction of 3 with $NO_3^{\bullet}$ generated from CAN photolysis

HPLC chromatogram of the raw reaction mixture

(recorded at a wavelength λ = 230 nm).

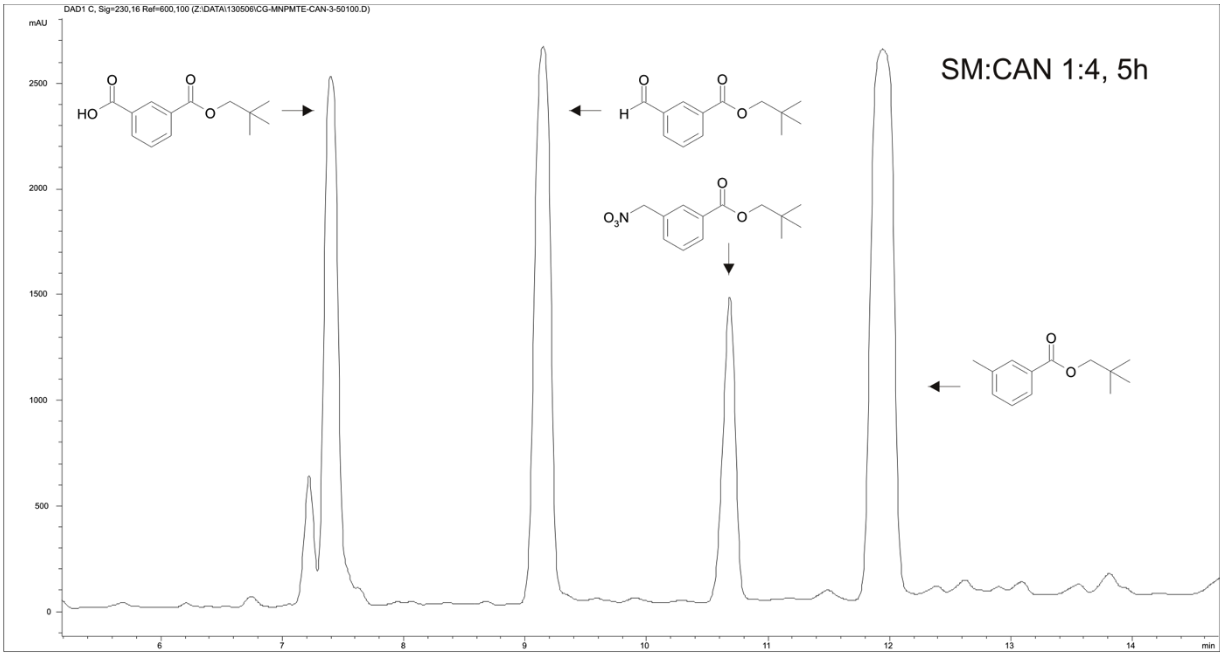

Reaction of 3 with $NO_3^{\bullet}$ generated from $NO_2^{\bullet}$ and O_3/O_2

HPLC chromatogram of the raw reaction mixture

(recorded at a wavelength λ = 230 nm).

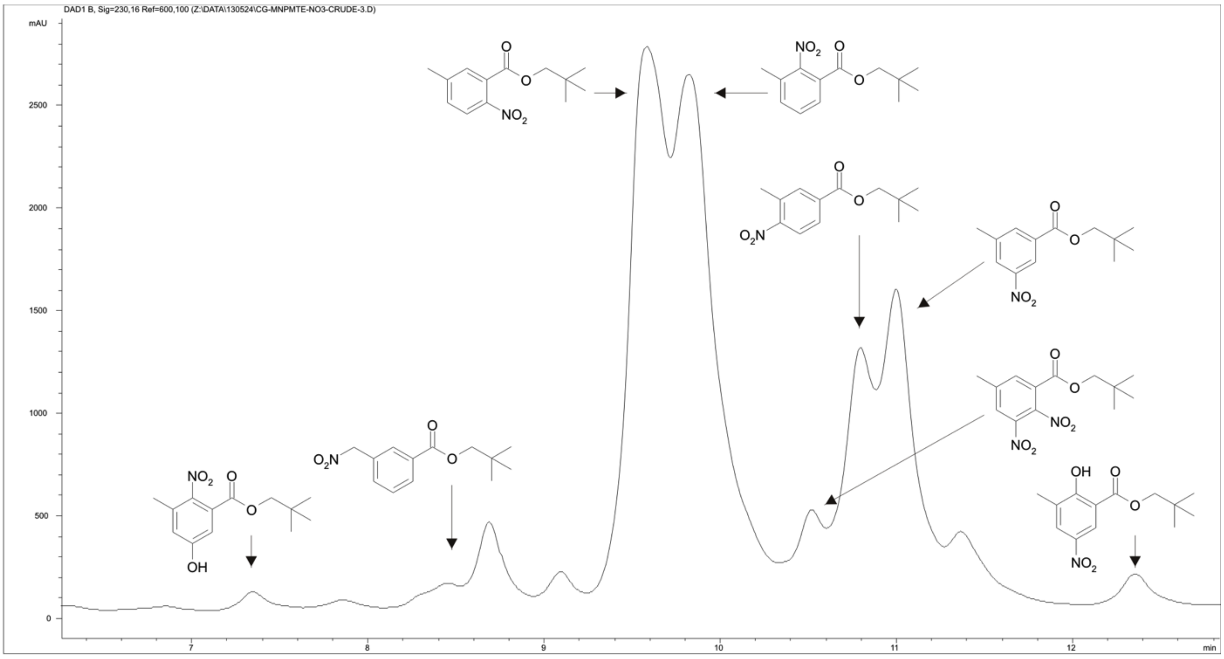

Supplement: File 1 — HPLC chromatograms of raw reaction mixtures. [file Beilstein_J_Org_Chem-09-1907-s001.pdf]
